# Nimbolide upregulates RECK by targeting miR-21 and HIF-1α in cell lines and in a hamster oral carcinogenesis model

**DOI:** 10.1038/s41598-017-01960-5

**Published:** 2017-05-17

**Authors:** Jaganathan Kowshik, Rajakishore Mishra, Josephraj Sophia, Satabdi Rautray, Kumaraswamy Anbarasu, G. Deepak Reddy, Madhulika Dixit, Sundarasamy Mahalingam, Siddavaram Nagini

**Affiliations:** 1 Department of Biochemistry and Biotechnology, Faculty of Science, Annamalai University, Annamalainagar, 608 002 Tamil Nadu India; 2 Centre for Life Sciences, School of Natural Sciences, Central University of Jharkhand, Ratu-Lohardaga Road, Brambe, Ranchi, 835205 Jharkhand India; 3 Medicinal Chemistry Research Division, Vishnu Institute of Pharmaceutical Education and Research, Narsapur, India; 4 Department of Biotechnology, Indian Institute of Technology Madras, Chennai, Tamil Nadu India

## Abstract

Reversion-inducing cysteine-rich protein with Kazal motifs (RECK), a potent inhibitor of matrix metalloproteinases (MMPs) is a common negative target of oncogenic signals and a potential therapeutic target for novel drug development. Here, we show that sequential RECKlessness stimulates angiogenesis and Notch signalling in the 7,12-dimethylbenz[a]anthracene (DMBA)-induced hamster buccal pouch (HBP) carcinogenesis model, a paradigm for oral oncogenesis and chemointervention. We also report the chemotherapeutic effect of nimbolide, a limonoid from the neem tree (*Azadirachta indica*) based on the upregulation of RECK as well as modulation of the expression of key molecules involved in invasion and angiogenesis. We demonstrate that nimbolide upregulates RECK by targeting miR-21, and HIF-1α resulting in reduced MMP activity and blockade of VEGF and Notch signalling. Nimbolide reduced microvascular density, confirming its anti-angiogenic potential. Molecular docking analysis revealed interaction of nimbolide with HIF-1α. Additionally, we demonstrate that nimbolide upregulates RECK expression via downregulation of HIF-1α and miR-21 by overexpression and knockdown experiments in SCC4 and EAhy926 cell lines. Taken together, these findings provide compelling evidence that targeting RECK, a keystone protein that regulates mediators of invasion and angiogenesis with phytochemicals such as nimbolide may be a robust therapeutic approach to prevent oral cancer progression.

## Introduction

Reversion-inducing cysteine rich protein with Kazal motifs (RECK), a membrane bound glycoprotein that plays a pivotal role in remodelling the extracellular matrix (ECM) by regulating the activity of matrix metalloproteinases (MMPs) is a potent inhibitor of tumor invasion, metastasis and angiogenesis^1–3^. The RECK protein containing multiple epidermal growth factor-like (EGF-like) repeats and serine-protease inhibitor (SPI) motifs is anchored via the C-terminal glycosylphosphatidylinositol (GPI) to the cell membrane. RECK primarily inhibits MMP-2, and -9 as well as a-disintegrin and metalloproteinase (ADAM-10). RECK regulates Notch signalling, which plays a critical role in angiogenesis^4^. RECK is widely expressed in normal tissues and nonneoplastic cell lines, but its expression is frequently downregulated in several tumours and in fibroblasts transformed by various oncogenes. Hence, RECKlessness is considered a hallmark of cancer^5^.

RECK downregulation is reported to stimulate invasion and angiogenesis in several tumours including liver, lung, breast, prostate, oral and digestive tract cancers^4–6^. The RECK gene is a common negative target of oncogenic signals as well as histone deacetylase (HDAC) that act on the binding site of the transcription factor Sp1 on the RECK gene promoter^7^. In addition, hypoxia and groups of miRs also cause transcriptional repression of RECK gene expression leading to upregulation of MMPs and ECM degradation^2, 8, 9^. Several synthetic and natural agents upregulate RECK through different strategies thereby blocking tumour progression. Overexpression of RECK has been demonstrated to inhibit tumour development and progression by downregulating the expression of MMPs and abrogating vascular endothelial growth factor (VEGF) and Notch signalling^5^. Thus, RECK may act as a promising prognostic marker and a potential therapeutic target for novel drug development. However, this needs to be tested in a well-established animal efficacy model for chemointervention.

The hamster buccal pouch (HBP) carcinogenesis model is one of the most well characterized animal tumour models for analyzing the stepwise evolution of oral cancer, the sixth most common cancer worldwide. HBP carcinomas induced by 7,12-dimethylbenz[a]anthracene (DMBA) share similarities with human oral squamous cell (OSCC) carcinomas in histology, metastatic dissemination and expression of a spectrum of molecular markers^10, 11^. We have extensively used this model to understand aberrant signalling pathways in OSCC and to test the chemopreventive and therapeutic potential of structurally diverse phytochemicals from the diet and medicinal plants^11–14^. In particular, limonoids from *Azadirachta indica*, commonly known as neem have emerged as promising anticancer agents based on preclinical studies in the HBP model^15, 16^. Accumulating evidence indicates that nimbolide, a major limonoid constituent of neem leaves modulates various signaling molecules and networks in a panel of cancer cells *in vitro* and animal tumour models *in vivo* ^16–23^. Recently, we demonstrated that nimbolide activates glycogen synthase kinase-3β to inhibit cell proliferation and induce apoptosis^24^.

In the present study, we analyzed changes in the expression of RECK and associated Notch and VEGF signaling during the sequential progression of HBP carcinomas from hyperplasia through dysplasia and papillomas. We also report the chemotherapeutic potential of nimbolide based on upregulation of RECK as well as modulation of the expression of key molecules involved in tumour invasion and angiogenesis. Further, we provide evidence to demonstrate that nimbolide activates RECK by inhibiting miR-21 and HIF-1α under hypoxic conditions in a cellular context using the oral cancer cell lines SCC131, SCC4 and the endothelial cell line EAhy926.

## Results

### RECK downregulation is associated with overexpression of MMPs and proangiogenic molecules during sequential progression of HBP carcinomas

**Figure 1:**
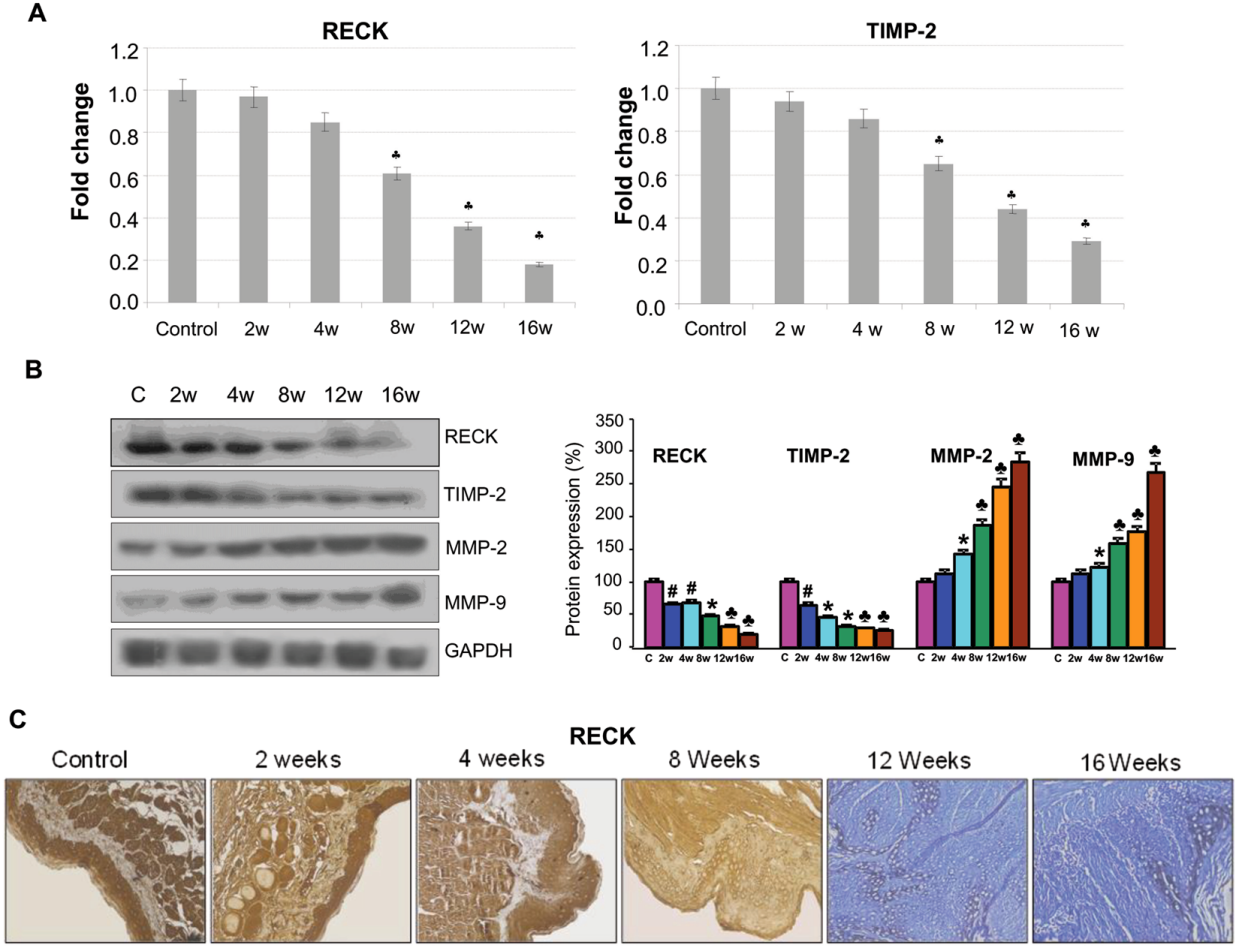
RECK is downregulated during sequential progression of HBP carcinoma. (**A**) qPCR analysis shows that RECK and TIMP-2 are downregulated during sequential progression of cancer. Data are the mean ± SD of three independent experiments (N = 3). ^♣^p < 0.001, *p < 0.01, ^#^p < 0.05 versus control. (**B**) Immunoblotting was performed to analyse the expression of RECK, TIMP-2, MMP-2 and MMP-9 in buccal pouch tissues. Data are the mean ± SD of three independent experiments (N = 3). ^♣^p < 0.001, *p < 0.01, ^#^p < 0.05 versus control. (**C**)Immunohistochemical staining of RECK (20X).

To determine the time-point at which RECK is inactivated during the stepwise evolution of HBP carcinomas, we first sought to explore RECK expression during the sequential progression of HBP tumours from normal buccal pouch epithelium. Quantitative RT-PCR, immunoblot and IHC analyses revealed a sequential decrease in the expression of RECK during the stepwise progression from hyperplasia through dysplasia to SCC with significant downregulation from the 12th week of DMBA application (Fig. 1). RECK expression showed a more significant inverse correlation with MMP-2 and MMP-9 compared to TIMP-2. Furthermore, the changes in RECK were more significant and pronounced relative to TIMP-2 indicating that it is a more reliable marker for tumour invasion.

**Figure 2:**
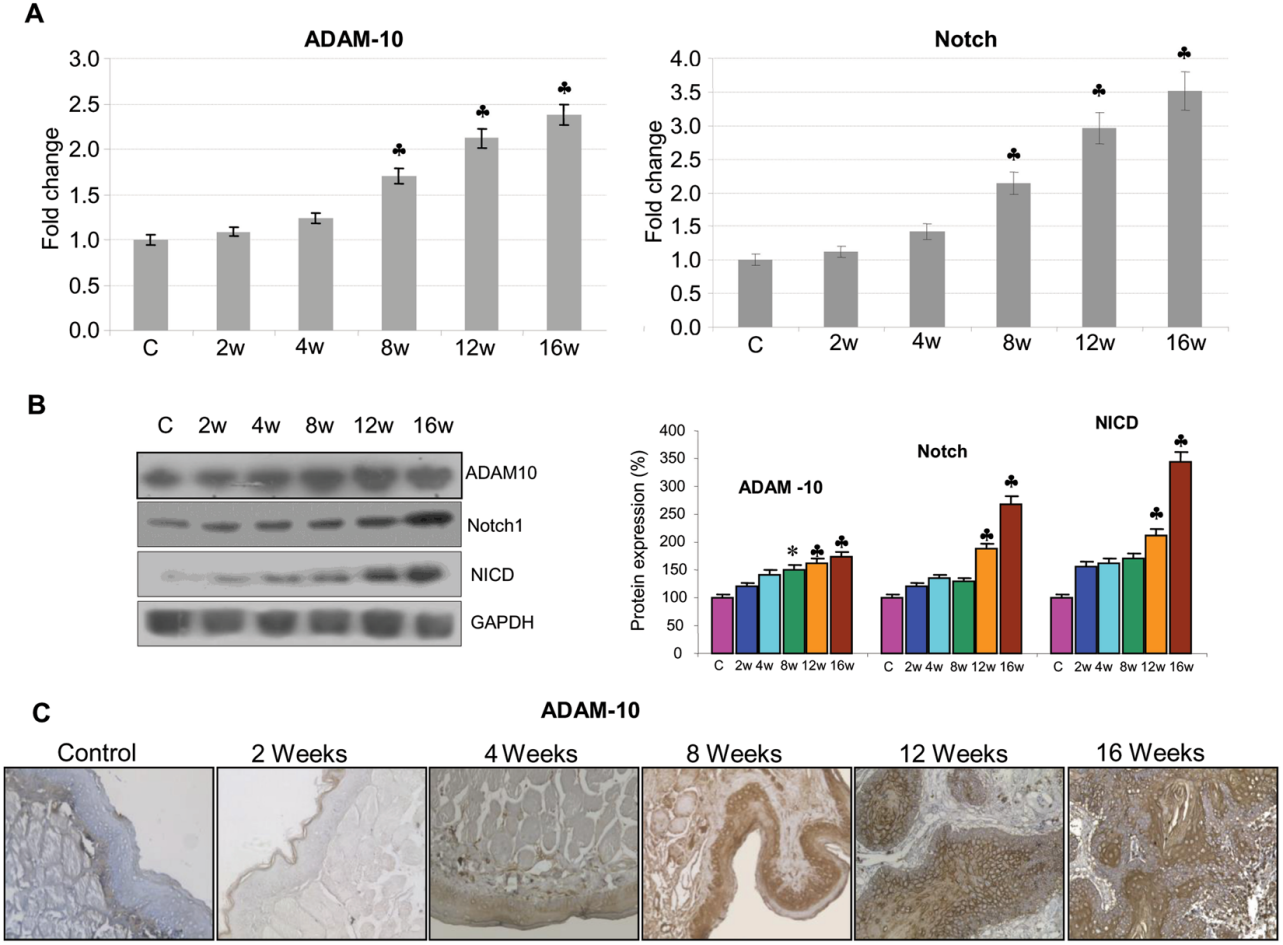
RECK downregulation activates Notch signalling. (**A**) qPCR analysis suggests that ADAM-10 and Notch are upregulated during sequential progression of cancer. Data are the mean ± SD of three independent experiments (N = 3). ^♣^p < 0.001, *p < 0.01, ^#^p < 0.05 versus control. (**B**) Protein samples were resolved on SDS-PAGE and western blot analysis was performed using antibodies against ADAM-10, Notch and NICD. GAPDH was used as loading control. Data are the mean ± SD of three independent experiments (N = 3). ^♣^p < 0.001, *p < 0.01, ^#^p < 0.05 versus control. (**C**) Immunohistochemical staining of ADAM-10. Images are shown in 20X magnification.

RECK is known to be a potent inhibitor of ADAM-10, a key component of Notch signalling. We next investigated whether RECK downregulation is linked to changes in the expression profile of key players in Notch signaling. We observed a sequential increase in the expression of ADAM-10, Notch1 and NICD in hamsters painted with DMBA compared to control, suggesting that loss of RECK activates Notch signaling (Fig. 2).

**Figure 3:**
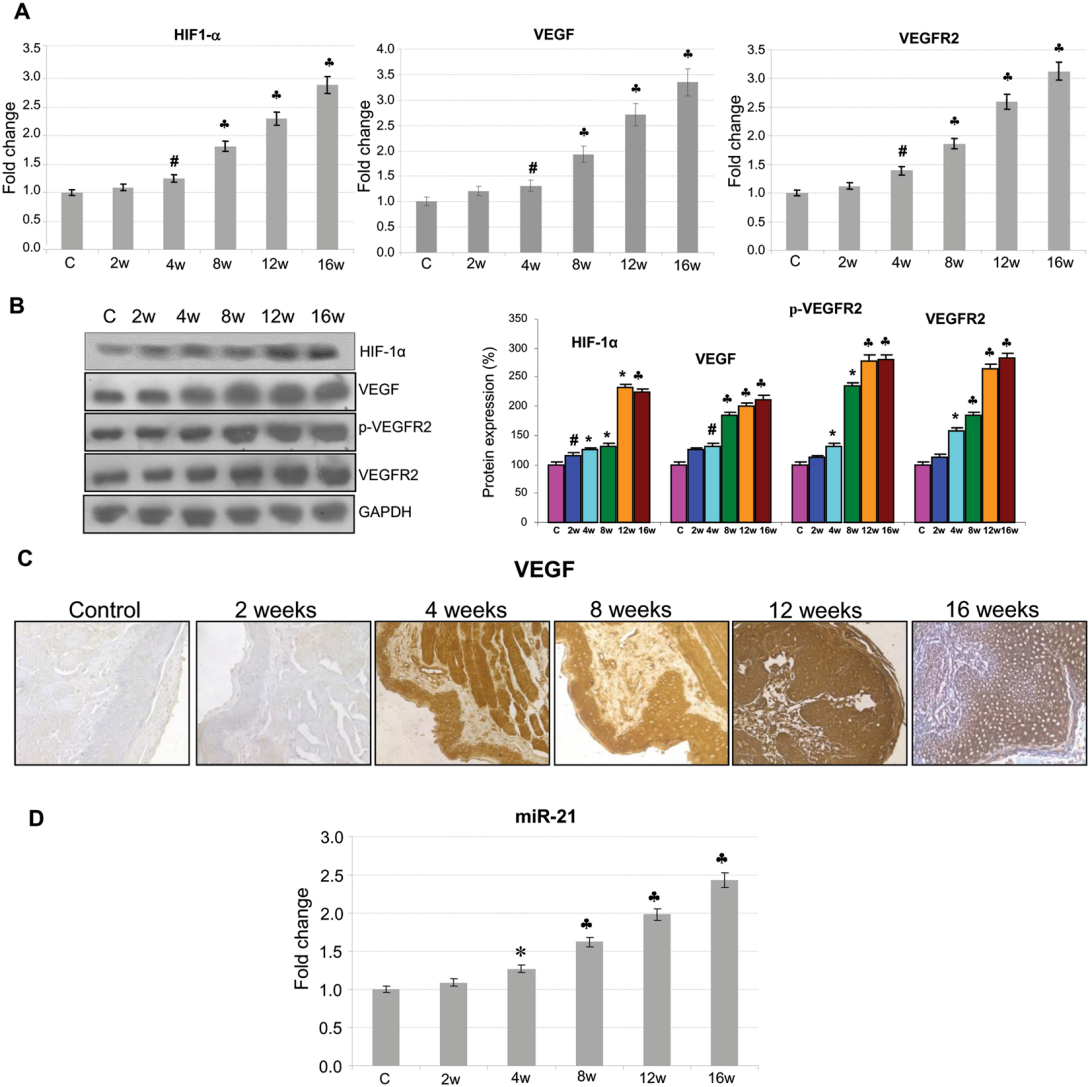
RECK downregulation activates VEGF signalling. (**A**) Transcript expression level of HIF-1α, VEGF and VEGFR2 determined by quantitative RT-PCR (N = 3). ^♣^p < 0.001, *p < 0.01, ^#^p < 0.05 versus control. (**B**) Protein samples were resolved on SDS-PAGE and western blot analysis was performed by probing with corresponding antibodies. GAPDH was used as loading control. Data are the mean ± SD of three independent experiments (N = 3). p < 0.001, *p < 0.01, ^#^p < 0.05 versus control. (**C**) Immunohistochemical staining of VEGF (20X). (**D**) Transcript expression level of miR-21 by quantitative RT-PCR.

Since RECKlessness as well as activated Notch signalling favour angiogenesis, we examined changes in VEGF signalling during neoplastic transformation of the HBP. Analysis of transcript and protein expression revealed progressive increase in VEGF and VEGFR2 as well as pVEGFR2, the active form of the receptor, in DMBA painted animals compared to control. This was associated with sequential overexpression of HIF-1α indicating intratumoral hypoxia. We next evaluated the expression of miR-21, an oncomiR that directly inhibits RECK expression and promotes angiogenesis. We found a sequential increase in the expression of miR-21 during stepwise progression of HBP carcinomas, suggesting this as a possible mechanism for RECK downregulation (Fig. 3).

### Nimbolide upregulates RECK expression by suppressing miR-21 and HIF-1α in the HBP model

**Figure 4:**
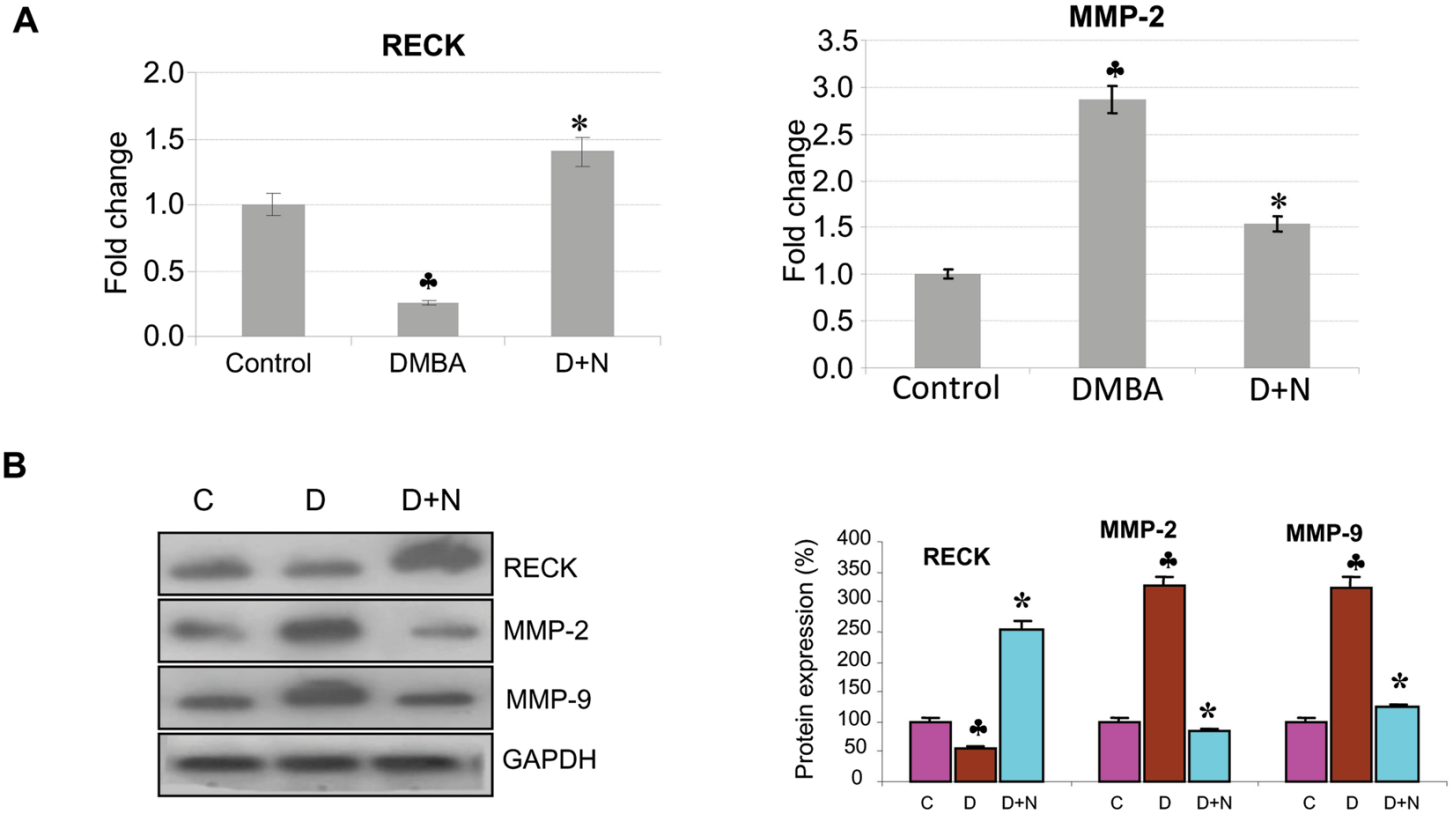
Nimbolide upregulates RECK expression. (**A**) qPCR analysis shows that RECK is upregulated, and MMP-2 was downregulated after nimbolide treatment (N = 3). (**B**) Immunoblotting was performed to analyse the expression of RECK, MMP-2 and MMP-9. Data are the mean ± SD of three independent experiments (N = 3). ^♣^p < 0.001 versus control. *p < 0.001versus DMBA. D+N indicates DMBA+ Nimbolide.

Since significant changes in transcript and protein expression of RECK and proangiogenic molecules were observed after 12 weeks of DMBA painting that correlated with tumour progression, we tested the chemotherapeutic efficacy of nimbolide at this time point. Details of the effects of nimbolide on tumour incidence have been described in our previous study^24^. Nimbolide administration significantly reduced tumour burden and delayed tumour growth by 52.4%^24^. This was associated with upregulation of RECK with simultaneous decrease in the expression of MMP-2 and MMP-9 (Fig. 4).

**Figure 5:**
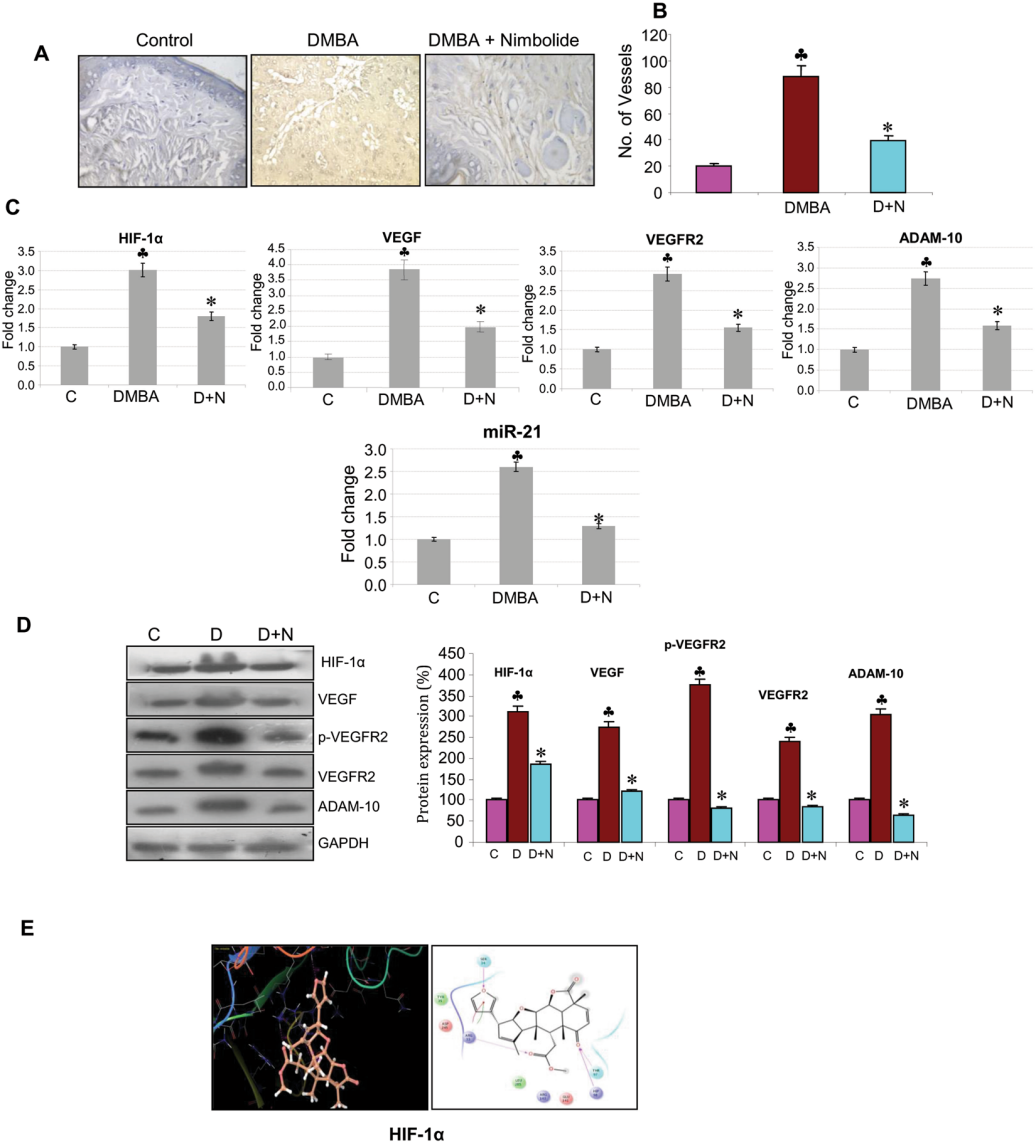
Nimbolide exerts anti-angiogenic effects. (**A**) Microvascular density analysis suggests that nimbolide treatment decreased vessel density (40X). (**B**) Bar graph representing number of vessels (N = 3). ^♣^p < 0.001 versus control. *p < 0.001 versus DMBA. (**C**) Transcript expression level of HIF-1α, VEGF, VEGFR2, ADAM-10 and miR-21 by quantitative RT-PCR. Data are the mean ± SD of three independent experiments. (**D**) Protein samples were resolved on SDS-PAGE and western blot analysis was performed by probing with corresponding antibodies. Data are the mean ± SD of three independent experiments (N = 3). (**E**) Plausible binding mode of nimbolide to HIF- 1α depicting interactions with Arg 33, His 98, Thr 99, Ser 34.

Analysis of microvascular density, an independent marker of tumour angiogenesis revealed decreased vascularity in nimbolide treated hamsters underscoring its anti-angiogenic potential (Fig. 5). Gene expression analyses suggest that nimbolide inhibits angiogenesis by targeting HIF1α and VEGF signalling. Furthermore, nimbolide downregulated ADAM-10, a key player in Notch signalling as well as miR-21.

Next, we performed molecular docking studies to investigate the possible interaction between nimbolide and HIF-1α, the negative regulator of RECK. Nimbolide was found to bind to HIF-1α with a docking score of −2.94. Nimbolide formed hydrogen bonds with Arg 33, His 98, Thr 99 and Ser 34 in the PAS-A domain of HIF-1α, suggesting regulatory effects on this molecule (Fig. 5E).

### Nimbolide upregulates RECK expression and downregulates key molecules involved in invasion and angiogenesis in OSCC and endothelial cell lines

**Figure 6:**
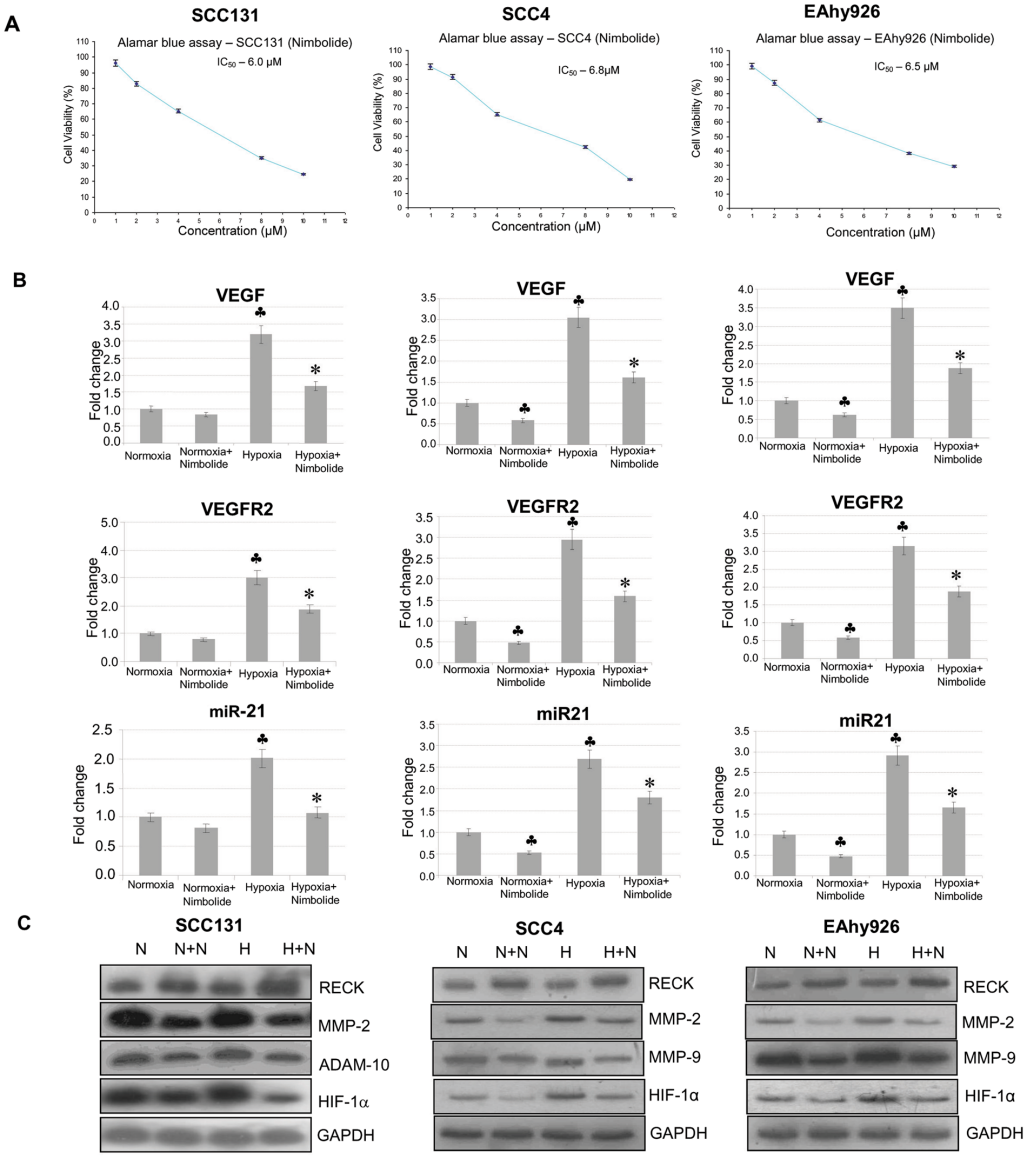
Nimbolide activates RECK and inhibits pro-invasive and pro-angiogenic molecules in a cellular context. (**A**) IC_50_ value for nimbolide in SCC131, SCC4 and EAhy926 cells (Alamar Blue assay). (**B**) Transcript expression level of VEGF, VEGFR2, and miR-21 by quantitative RT-PCR in SCC131, SCC4 and EAhy926 cell lines. Data are the mean ± SD of three independent experiments (N = 3). ^♣^p < 0.001 versus normoxia. *p < 0.001versus hypoxia. (**C**) Immunoblotting was performed to analyse the expression of RECK, MMP-2, ADAM-10 and HIF-1α in the absence or presence of nimbolide (1 µm) under normoxic and hypoxic conditions.

The inhibitory effects of nimbolide on tumour invasion and angiogenesis prompted us to examine whether nimbolide blocks invasion and angiogenesis in a cellular context. We first analysed the effect of varying concentrations of nimbolide on the viability of SCC131, SCC4 and EAhy926 cells. Nimbolide reduced the viability of these cell lines in a dose-dependent manner with an IC_50_ of 6, 6.8 and 6.5 µm respectively. As the viability of cells was 100% at 1 µM, this concentration was used for further experiments (Fig. 6A).

Since hypoxia is a potent stimulus for angiogenesis, we determined the expression of RECK, MMPs, HIF-1α and miR21 under normoxic and hypoxic conditions both in the presence and absence of nimbolide. Upregulation of RECK by nimbolide significantly correlated with downregulation of MMP-2, ADAM-10, HIF1α, VEGF and VEGFR2 in all the three cell lines studied under normoxic as well as hypoxic conditions (Fig. 6). miR-21, the negative regulator of RECK was significantly downregulated by nimbolide in all the cell lines tested.

### Nimbolide inhibits expression of pro-invasive and pro-angiogenic molecules in cells overexpressing RECK

**Figure 7:**
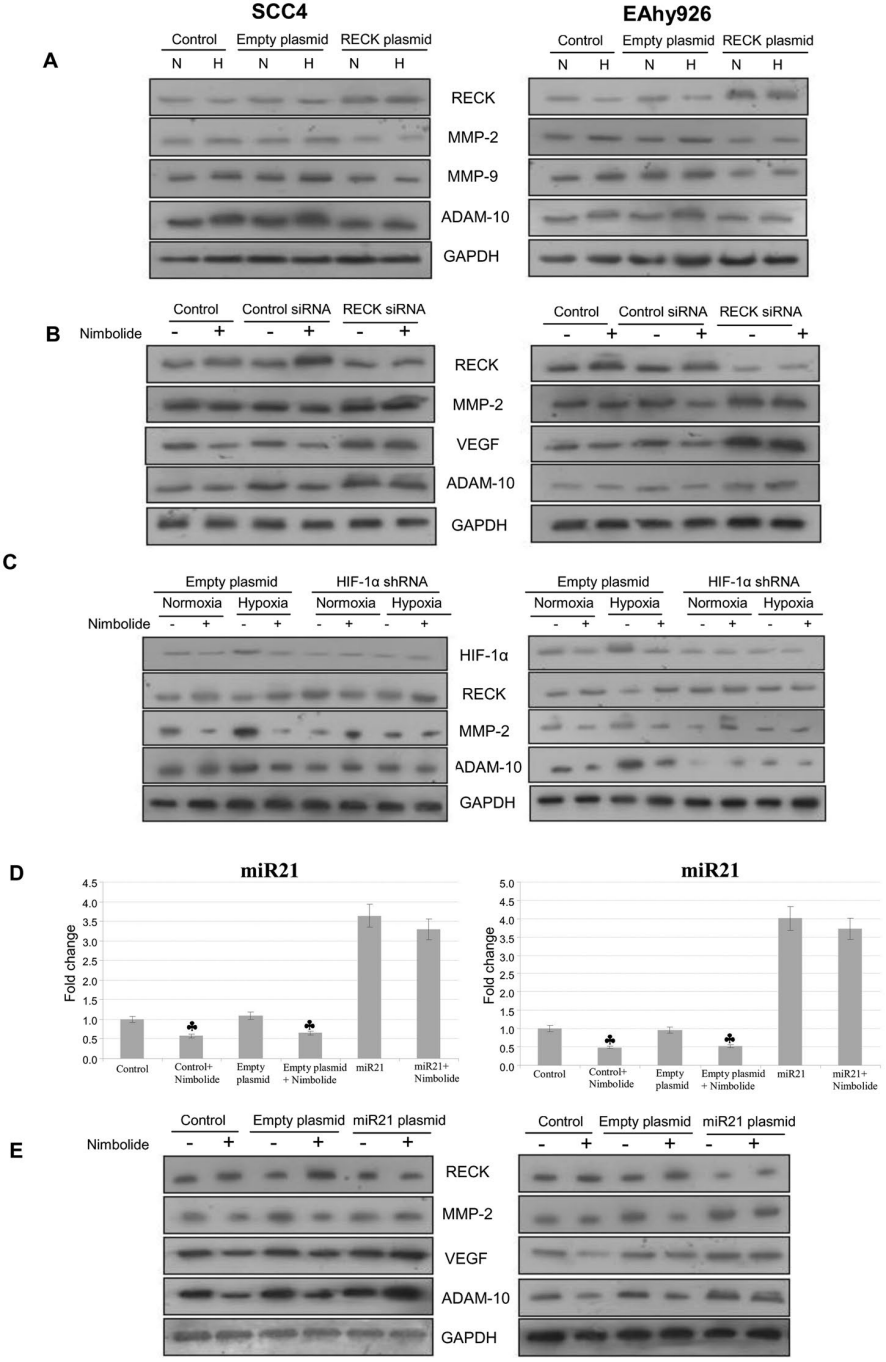
Nimbolide activates RECK by targeting HIF-1α and miR-21. (**A**) Immunoblot analyses of RECK, MMP-2, MMP-9 and ADAM-10 in control, empty plasmid and RECK plasmid transfected SCC4 and EAhy926 cells under normoxic and hypoxic conditions (N = 3). (**B**) Immunoblot analyses of RECK, MMP-2, VEGF and ADAM-10 in control, Control siRNA and RECK siRNA transfected cells in the absence or presence of nimbolide (1 µm). (**C**) Immunoblotting was performed to analyse the expression of HIF-1α, RECK, MMP-2 and ADAM-10 in empty vector and HIF-1α RNAi transfected cells under normoxic and hypoxic conditions in the presence or absence of nimbolide (1 µm). (**D**) Transcript expression level of miR-21 in control, empty plasmid and miR-21 plasmid in the presence or absence of nimbolide (1 µm) in SCC4 and EAhy926 cells as determined by quantitative RT-PCR. ^♣^p < 0.001 versus normoxia. *p < 0.001versus hypoxia. (**E**) Immunoblotting was performed to analyse the expression of RECK, MMP-2, VEGF and ADAM-10 in control, empty plasmid and miR-21 plasmid in the presence or absence of nimbolide (1 µm) in SCC4 and EAhy926.

To demonstrate that RECK inhibits MMP-2,-9 and ADAM-10, we transfected RECK plasmid in SCC131, SCC4 and EAhy926 cell lines and assessed RECK overexpression by immunoblotting. Since expression of RECK was low in SCC131 cells due to poor transfection efficiency, further experiments were performed in SCC4 and EAhy926 cells which showed higher RECK expression. RECK overexpression in SCC4 and EAhy926 cells were associated with significant downregulation of MMP-2,-9 and ADAM-10 under both normoxic and hypoxic conditions compared to control or empty vector transfected cells. The effects of hypoxia were significantly inhibited by RECK overexpression consistent with our findings that nimbolide inhibits MMPs and ADAM-10 via RECK activation (Fig. 7A).

To further strengthen these observations, we next silenced RECK using RECK siRNA in the presence and absence of nimbolide. SCC4 and EAhy926 cells were transfected with control or RECK siRNA as per manufacturer’s instruction and the knockdown confirmed by western blot analysis. Our results revealed that RECK inhibition significantly increased the expression of MMP-2, VEGF and ADAM-10 both in presence and absence of nimbolide. Together, these findings provide firm evidence that nimbolide inhibits MMP-2, VEGF and ADAM-10 via RECK activation (Fig. 7B). Furthermore, transfection with RECK plasmid/siRNA did not significantly alter the viability of SCC4 and EAhy926 cells (Supplementary Fig. S1).

### Nimbolide-induced RECK activation is associated with HIF-1α downregulation

To determine whether HIF-1α depletion impacts RECK expression, we transfected SCC4 and EAhy926 cells with empty or HIF-1α RNAi plasmid under normoxic and hypoxic conditions. HIF-1α knockdown was confirmed by western blot analysis. Silencing HIF-1α increased RECK expression with concomitant downregulation of MMP-2 and ADAM-10. Although nimbolide did not influence HIF-1α expression in HIF-1α silenced cells, addition of nimbolide to cells transfected with empty vector downregulated HIF-1α expression and upregulated RECK (Fig. 7C). In addition, we observed decreased miR-21 expression in HIF-1α knockdown cells (Supplementary Fig. S2). These findings indicate that nimbolide activates RECK by targeting HIF-1α.

### Nimbolide activates RECK by targeting miR-21

To ascertain inhibition of RECK by miR-21, we overexpressed miR-21 in the presence and absence of nimbolide. SCC4 and EAhy926 cells were transfected with empty plasmid or miR-21 plasmid using FuGENE. miR-21 overexpression was confirmed by qRT-PCR. We observed RECK downregulation with simultaneous increase in the expression of MMP-2, VEGF and ADAM-10 in miR-21 overexpressed cells, both in the presence and absence of nimbolide. In cells overexpressing miR-21, addition of nimbolide did not influence RECK expression. However, in both control and empty vector transfected cells, nimbolide reduced miR-21 expression accompanied by RECK overexpression. Taken together, these results demonstrate that nimbolide activates RECK by targeting miR-21 in these cell lines (Fig. 7D and E).

## Discussion

The extracellular matrix (ECM) with its complex and diverse network of proteins plays a central role in normal tissue homeostasis. Aberrant expression of MMPs breaks down the ECM leading to tumour invasion and angiogenesis. Accordingly, MMPs have emerged as strong *bona fide* cancer biomarkers while RECK, a potent MMP inhibitor has evolved as a reliable prognostic marker and potential therapeutic target^2, 25–27^. There is also growing evidence to indicate that activation of the Notch pathway facilitates tumour progression, and RECK inhibits ADAM-10 mediated Notch signaling^4, 28^.

We report for the first time progressive Recklessness that correlated with increasing MMP expression during stepwise evolution of HBP carcinomas and upregulation of RECK following chemointervention with nimbolide. Furthermore, nimbolide decreased transcript and protein levels of ADAM-10, a more efficient sheddase than MMPs. This in turn would serve to stabilize the interaction between Notch ligands and their receptors thereby regulating Notch signaling. Significantly, downregulation of MMPs and ADAM-10 in RECK overexpressed but not in RECK silenced SCC4 and EAhy926 cells supports our premise that nimbolide inhibits MMPs and ADAM-10 via RECK activation.

In addition to ECM degradation, formation of new blood vessels is an essential prerequisite for tumour progression^29^. Using multiple xenograft mouse models, functional genomics and biochemical assays, Walsh *et al*. unequivocally showed that RECK suppresses neoangiogenesis by regulating VEGF signaling^1^.On the other hand, hypoxia, a potent stimulus for angiogenesis downregulates RECK by recruiting HIF1-α and HDAC-1 to the reverse hypoxia responsive element (rHRE2) site in the promoter^7^. Binding of HDAC-1 to the ODD domain of HIF-1α stabilizes it leading to nuclear translocation and transactivation of hypoxia responsive genes such as VEGF^30^. In the present study, we observed a significant correlation between sequential loss of RECK gene expression and angiogenesis as reflected by enhanced expression of key components of VEGF signaling and increased microvascular density in the hamster buccal pouch that was ameliorated by nimbolide treatment. Additionally, experiments in OSCC and EAhy926 cells also showed that nimbolide downregulates VEGF and VEGFR2 under hypoxic conditions. Furthermore, both HIF-1α knockdown and nimbolide treatment induced RECK overexpression suggesting that HIF-1α is a likely target of nimbolide. This was potentiated by molecular docking studies that revealed interaction of nimbolide with HIF-1α in the PAS-A domain, which is essential for its transcriptional activity and interaction with HIF-1β.

RECK, an endogenous inhibitor of proteins that promote tumor invasion and angiogenesis is a negative target for miR-21, an oncomiR that binds directly to the 3′-UTR of RECK^8, 31^. The sequential increase in miR-21 expression from hyperplasia through dysplasia to SCC together with hypoxia may act cooperatively to downregulate RECK in the hamster buccal pouch in the present study. Overexpression of miR-21 documented extensively in a wide range of malignant tumours simultaneously regulates cell proliferation, migration, apoptosis, invasion, and angiogenesis^32, 33^. miR-21 exerts pleiotropic roles in tumour development and progression by activating oncogenic signaling pathways and transcription factors^33, 34^. A positive correlation was observed between expressions of Notch-1 and miR-21 in colorectal cancer development suggesting a possible crosstalk^35^. RECK control by miR-21 overexpression results in invasion and tumour recurrence in patients following surgical treatment of prostate cancer^8^.

**Figure 8:**
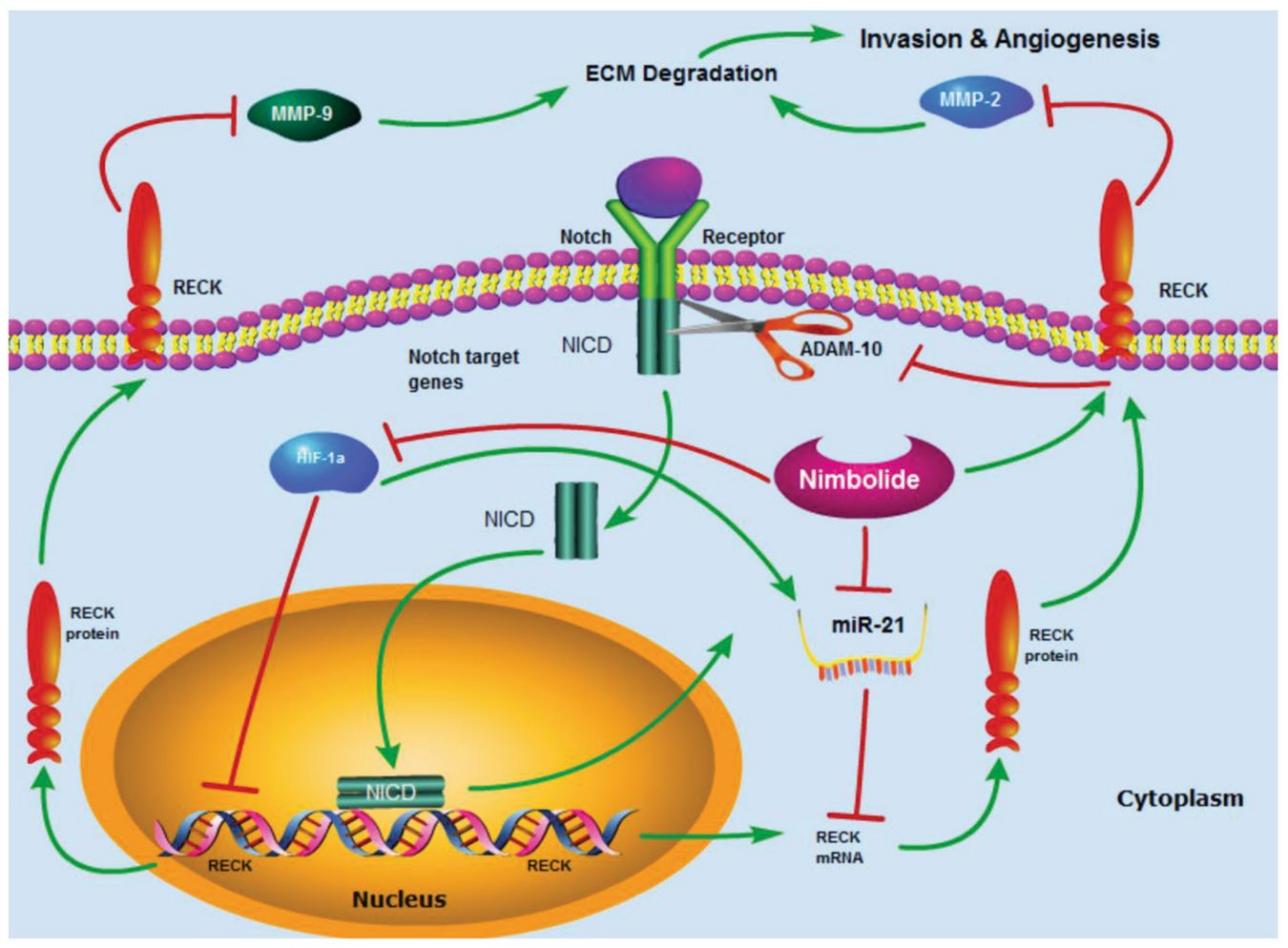
Schematic representation of the mechanism of action of nimbolide. Dietary administration of nimbolide activates RECK and prevents ECM degradation, and blocks activation of Notch signalling and HIF-1α mediated VEGF signalling thereby inhibiting angiogenesis. Nimbolide also inhibits miR-21, a negative regulator of RECK. Nimbolide prevents cancer progression by modulating the expression of genes and pathways involved in invasion and angiogenesis as well as by targeting miR-21.

Various phytochemicals that inhibit invasion and angiogenesis have been shown to suppress miR-21 with concomitant upregulation of RECK^36–38^. Recently, Li *et al*.^39^ demonstrated that iicarin, an active ingredient of the Chinese medicinal plant Epimedium regulates proliferation and apoptosis of human ovarian cancer cells by targeting miR-21 and substantially increasing RECK protein expression. Curcumin was shown to inhibit the growth of oesophageal cancer cell lines by inhibiting Notch signaling and Notch-specific miR-21^40^. Nimbolide activates RECK by functioning as an antagonist of miR-21 with consequent reduced MMP activity and blockade of VEGF and Notch signaling. Although nimbolide activated RECK by downregulating miR-21 in the hamster model and in empty vector transfected cells it failed to exert the same effect in SCC4 and EAhy926 cells overexpressing miR-21, presumably due to lower concentration (1 µm) used in the present study. Further studies are warranted to find the exact mechanism of miR-21 inhibition by nimbolide. Figure 8 summarizes the mechanism of the chemotherapeutic action of nimbolide in the HBP model.

Taken together, these findings provide compelling evidence that RECK is a keystone protein that regulates mediators of tumour invasion and angiogenesis. Our results also indicate that targeting miR-21 and HIF-1α with phytochemicals such as nimbolide may be a robust therapeutic approach to modulate multiple proteins and pathways deregulated in oral cancer.

## Materials and Methods

### Chemicals

Chemical reagents were obtained from Sigma Chemical Company (St. Louis, MO, USA) and HiMedia Labs (Mumbai, India). Nimbolide was purchased from Asthagiri Herbal Research Foundation, Chennai. Antibodies for GAPDH, TIMP-2, RECK, MMP-2, MMP-9, VEGF, VEGFR2 and HIF1α were purchased from Santa Cruz Biotechnology, USA. p-VEGFR2^Tyr1175^, Notch-1, NICD, ADAM-10 and histone (H2B) antibodies were from Cell Signaling Technology, USA. Fetal bovine serum was from GIBCO, Invitrogen, NY, USA. Power SYBR Green PCR master mix was obtained from Applied Biosystems, California, USA. FuGENE transfection reagent was procured from Promega. Oligonucleotide primers were procured from Sigma Genosys, San Ramon, USA. All other reagents used were of analytical grade.

### Animals and Diet

Eight to ten weeks old male Syrian hamsters weighing between 100–110 g were used in this study. The animals were obtained from the National Centre for Laboratory Animal Sciences, Hyderabad, India. The animals were housed three to a cage and provided with standard pellet diet and water ad libitum. The protocols for the animal experiments were approved by the Institutional Animal Ethics Committee, Annamalai University and conducted according to the guidelines laid down by the Committee for the Purpose of Control and Supervision on Experiments on Animals (CPCSEA).

## Experimental Design

### Experiment 1

The animals were randomized into experimental and control groups and divided into six groups of six animals each. Hamsters in group 1 received basal diet alone and served as control. The right buccal pouches of hamsters in the experimental groups 2–6 were painted with a 0.5% solution of DMBA in liquid paraffin using a number four brush, three times per week for 2, 4, 8, 12, and 16 weeks, respectively^10, 41^. At the end of the specified experimental period, animals in each group were sacrificed by cervical dislocation after an overnight fast. The buccal pouch tissues were immediately subdivided and processed for distribution to each experiment.

### Experiment 2

The animals were randomized into experimental and control groups and divided into 3 groups of 6 animals each. In group 1, the right buccal pouches of hamsters were painted with 0.5% DMBA in liquid paraffin three times a week for 12 weeks. In group 2, animals received 100 µg/ kg bw nimbolide dissolved in 0.5% DMSO daily from 12^th^ till 16^th^ week^24^. Group 3 animals received basal diet alone and served as untreated controls. The experiment was terminated after the 16^th^ week and all animals were sacrificed by cervical dislocation after an overnight fast.

### Experiment 3

EAhy926 and SCC131 cell lines were cultured in DMEM basal medium with 10% fetal bovine serum with antibiotics. SCC4 cells were maintained in DMEM/F12 supplemented with 10% fetal bovine serum, 2 mM Glutamine and 0.4 µg/ml hydrocortisone. Confluent cultures of EAhy926, SCC131 and SCC4 cells were subcultured and maintained in a CO_2_ incubator at 37 °C. For hypoxic condition, cells were incubated in a hypoxia chamber with 1% O_2_.

### RNA extraction and quantitative real-time RT-PCR

Total RNA from the buccal pouch tissues was extracted using Trizol reagent as described previously^42^. The integrity of RNA was checked before being used for reverse transcription. 5 µg of isolated total RNA was reverse-transcribed to cDNA in a reaction mixture containing 4 µl of 5 X reaction buffer, 2 µl of dNTP mixture (10 mM), 20 units of RNase inhibitor, 200 units of avian-myeloblastosis virus (AMV) reverse transcriptase and 0.5 µg of oligo(dT) primer (Promega Corporation, WI, USA) in a total volume of 20 µl. The reaction mixture was incubated at 42 °C for 60 min and the reaction terminated by heating at 70 °C for 10 min. The cDNA was stored at −80 °C until further use.

Quantitative RT-PCR was performed using Power SYBR Green master mix according to the manufacturer’s instructions using a StepOne Plus thermocycler (Applied Biosystems). To the 1 × PCR master mix, 2.5 µl of each cDNA was added in a 20 µl final volume. The PCR conditions were as follows: 95 °C for 5 min, 40 cycles of 30 s at 95 °C, 30 s at 52 to 60 °C (based on the target), and 60 s at 72 °C. All reactions were carried out in triplicate, and the relative quantitative fold change compared to control was calculated using the comparative Ct method.

### miRNA isolation

miRNA was isolated using miRNeasy mini kit method. miRNA was quantified by Biophotometer at 260 and 280 nm absorbance. cDNA was synthesized using Ncode VILO miRNA cDNA synthesis kit following the manufacturer’s instructions. Expression levels of miR-21 were quantified using StepOne Plus thermocycler (Applied Biosystems).

### Western blotting

Proteins were extracted from buccal pouch tissues and cell lines using lysis buffer containing 62.5 mM Tris (pH 6.8), 10% SDS, 5% 2-mercaptoethanol, 10% glycerol and bromophenol blue. Equal amount of protein extracts were loaded and resolved using SDS-PAGE and transferred to nitrocellulose membranes. The blots were then incubated for 1 h in 1X PBS containing 5% non-fat dry milk. The membranes were then probed with primary and secondary antibodies as per manufacturer’s instructions. The proteins were visualized using enhanced chemiluminescence. Densitometry was performed on IISP flat bed scanner and quantitated with Total Lab 1.11 software.

### Immunohistochemistry

Paraffin embedded tissue sections were deparaffinised, rehydrated and subjected to antigen retrieval and endogenous peroxidase blocking. The sections were then incubated with RECK, HIF-1α, VEGF and ADAM-10 rabbit polyclonal antibodies at room temperature for 3 h. The slides were washed with TBS and then incubated with biotin-labeled secondary antibody followed by streptavidin–biotin–peroxidase (Dako, Carprinteria, CA, USA) for 30 min each at room temperature. Tissue slides were stained with DAB chromogen and counterstained with hematoxylin followed by dehydration through increasing ethanol dilutions ending in a xylene bath. The stained sections were then photographed using an Inverted Fluorescent Microscope (Leica Microsystem Vertrieb GmbH, Wetzler, Germany) attached with digital camera DFC295.

### Microvascular density (MVD)

Microvascular density was assessed by immunohistochemical staining with anti-CD34 antibody. The areas of highest neovascularization were located and the images captured in a minimum of five different fields. Two independent scientists performed the vessel density evaluation and the average was regarded as the final microvessel count and represented as number of vessels/field of view.

### Molecular docking

The interactions of nimbolide with HIF-1α was identified using Glide XP docking algorithm of Schrodinger Suite. Digital structure of HIF-1α was retrieved from PDB website (PDB ID: 3OD4). The obtained structure was processed on satisfying valency by adding hydrogen atoms or removing unbound water. Energy minimization was performed using OPLS-2005 force field. Later, binding pockets of each protein were identified using SITEMAP tool of Schrodinger suite and for the highest volume site, a grid was generated, and previously energy minimized nimbolide was docked. Post-docking calculations were performed in order to calculate the binding energy of nimbolide with respective proteins.

### Alamar blue assay

Cell viability was measured by Alamar Blue assay. Briefly, Alamar blue (resazurin sodium salt from Sigma) was dissolved in phosphate buffered saline pH 7.4 and a final working concentration of 0.1 mg/mL was used. Resazurin is a redox indicator, which measures the reducing environment of the cell by reducing to a highly fluorescent resorufin. Nimbolide at different concentrations (1, 2, 3, 4, 5, 6, 12, 24 µM) was added to the cells and after 24 h, Alamar blue was added and the plates were incubated at 37 °C for 4 h. The colour change was monitored colorimetrically at 595 nm and 570 nm to evaluate oxidized versus reduced forms respectively of the reagent by using Bio-Rad Multimode Plate Reader.

### Plasmid construction

RECK open reading frame was amplified from a PBMC cDNA library using appropriate primers and cloned into pcDNA3 vector as a C- terminal enhanced green fluorescent protein (eGFP) fusion in Kpn 1 and Xho 1 sites. miR-21 (#21114) and HIF-1α RNAi (#21103) plasmids were obtained from Addgene, USA. The integrity of all the constructs was confirmed by DNA sequencing.

### Transfection

Plasmid transfection was performed using FuGENE transfection reagent according to the manufacturer’s instructions. Control or RECK-siRNA (Sigma Chemical Company, St. Louis, MO, USA), was transfected with FuGENE according to the manufacturer’s instructions. Briefly, SCC4 and EAhy926 cells were transfected using FuGENE at 60–70% confluency. Cells were incubated with Plasmid/siRNA and FuGENE complex for 8 h. After 8 h, the media was renewed followed by treatment with nimbolide for 24 hours. At the end of 48 h, RNA and proteins were isolated from the cells for further analysis.

### Statistical analysis

The data are expressed as Mean ± Standard Deviation (SD). Statistical analysis was performed using one-way ANOVA followed by Dunnett’s test for *in vivo* and Tukey posthoc test for the *in vitro* experiments. A probability value of less than 0.05 was considered significant.

## Electronic supplementary material

Supplementary figures
