# Supplementary material for: Nimbolide upregulates RECK by targeting miR-21 and HIF-1α in cell lines and in a hamster oral carcinogenesis model
Source: Sci Rep. 2017 May 17;7:2045. doi: 10.1038/s41598-017-01960-5 (PMC5435722; doi:10.1038/s41598-017-01960-5)
Supplement: Supplementary file 1 — Supplementary figures [file 41598_2017_1960_MOESM1_ESM.pdf]

Nimbolide upregulates RECK by targeting miR-21 and HIF-1α in cell lines and in a hamster oral carcinogenesis model

Jaganathan Kowshik[1], Rajakishore Mishra[2], Josephraj Sophia[1], Satabdi Rautray[1], Kumaraswamy Anbarasu[4], G Deepak Reddy[3], Madhulika Dixit[4], Sundarasamy Mahalingam[4], Siddavaram Nagini[1]*

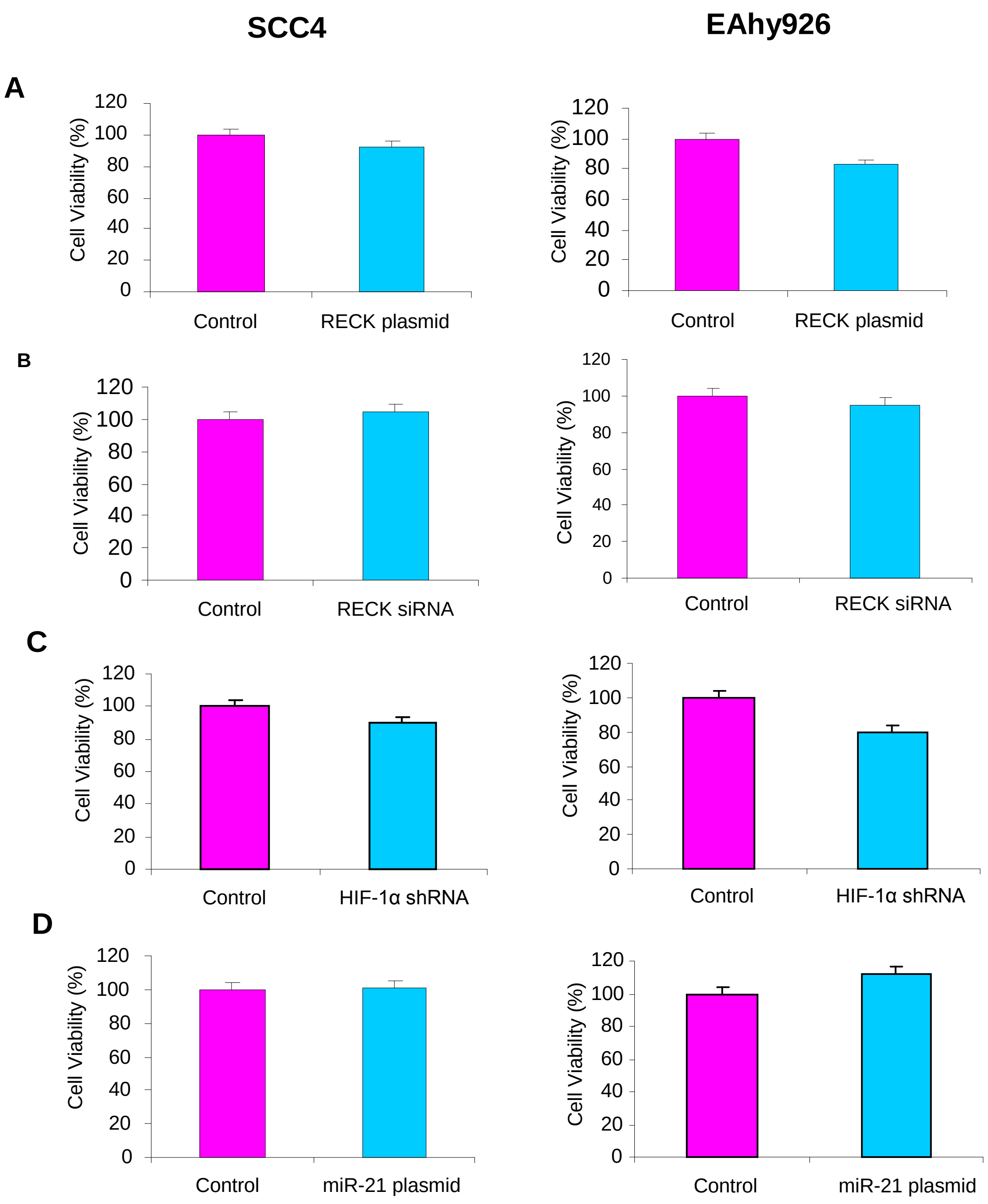
SCC4
EAhy926
A
B
C
D
Cell Viability (%)
120
100
80
60
40
20
0
Control
RECK plasmid
RECK siRNA
HIF-1α shRNA
miR-21 plasmid

A

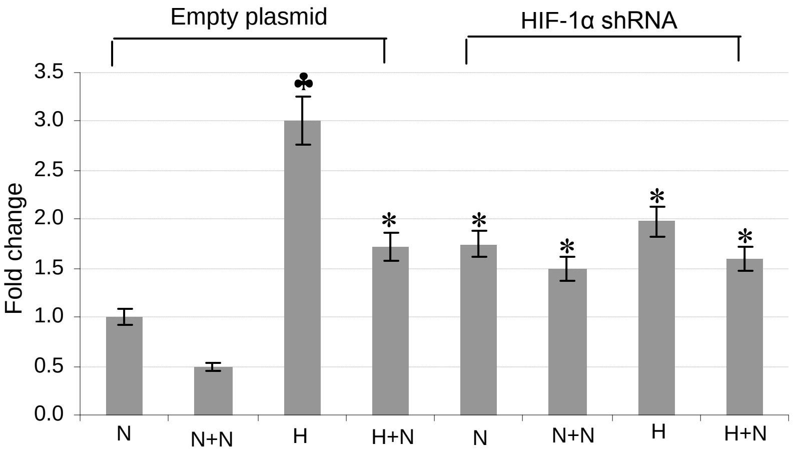
Empty plasmid
HIF-1α shRNA
Fold change
3.5
3.0
2.5
2.0
1.5
1.0
0.5
0.0
N
N+N
H
H+N
N
N+N
H
H+N

B

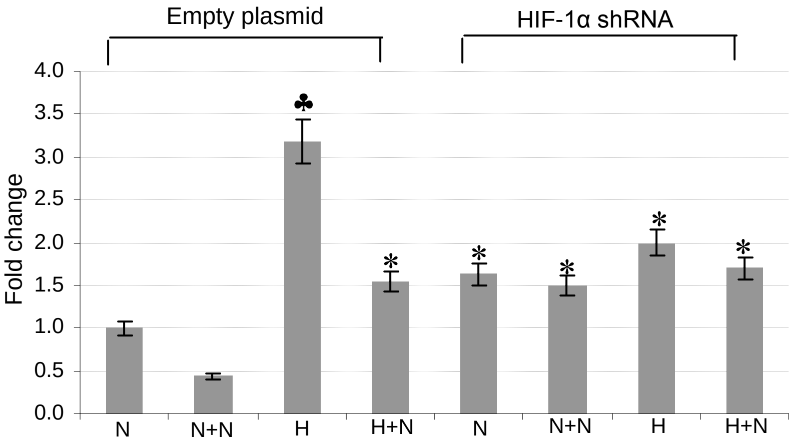
Empty plasmid
HIF-1α shRNA
Fold change
4.0
3.5
3.0
2.5
2.0
1.5
1.0
0.5
0.0
N
N+N
H
H+N
N
N+N
H
H+N

Supplementary Figure S1. Effect of RECK plasmid, siRNA, HIF-1α shRNA and miR-21 plasmid transfection on cell viability in SCC4 and EAhy926 cells.

A-D Viability of control and RECK plasmid, siRNA, HIF-1α shRNA and miR-21 plasmid transfected SCC4 and EAhy926 cells.

Supplementary Figure S2. Nimbolide inhibits miR-21 expression.

A & B Transcript expression level of miR-21 in empty vector and HIF-1α RNAi transfected cells in the presence or absence of nimbolide (1µm) in SCC4 and EAhy926 cells as determined by quantitative RT-PCR.

N - Normoxia, H - Hypoxia, N+N - Normoxia + Nimbolide, H+N – Hypoxia + Nimbolide

♣ $p<0.001$ versus normoxia

* $p<0.001$versus hypoxia
